# Environmental isolation explains Iberian genetic diversity in the highly homozygous model grass *Brachypodium distachyon*

**DOI:** 10.1186/s12862-017-0996-x

**Published:** 2017-06-15

**Authors:** Isabel Marques, Valeriia Shiposha, Diana López-Alvarez, Antonio J. Manzaneda, Pilar Hernandez, Marina Olonova, Pilar Catalán

**Affiliations:** 1 Departamento de Ciencias Agrarias y del Medio Natural, Escuela Politécnica Superior de Huesca, Universidad de Zaragoza, Ctra. Cuarte km 1, 22071 Huesca, Spain; 2 Department of Botany, Institute of Biology, Tomsk State University, Lenin Av. 36, Tomsk, 634050 Russia; 3 Departamento de Biología Animal, Biología Vegetal y Ecología, Universidad de Jaén, Paraje Las Lagunillas s⁄n, 23071 Jaén, Spain; 4 Instituto de Agricultura Sostenible (IAS-CSIC), Alameda del Obispo s/n, 14004 Córdoba, Spain; 5 Present address: Centro de Bioinformática y Biología Computacional de Colombia, BIOS, Parque los Yarumos, Manizales, Colombia

**Keywords:** *Brachypodium*, Environmental isolation, Genetic diversity, Homozygosis, Selfing, Soil pH

## Abstract

**Background:**

*Brachypodium distachyon* (Poaceae), an annual Mediterranean Aluminum (Al)-sensitive grass, is currently being used as a model species to provide new information on cereals and biofuel crops. The plant has a short life cycle and one of the smallest genomes in the grasses being well suited to experimental manipulation. Its genome has been fully sequenced and several genomic resources are being developed to elucidate key traits and gene functions. A reliable germplasm collection that reflects the natural diversity of this species is therefore needed for all these genomic resources. However, despite being a model plant, we still know very little about its genetic diversity. As a first step to overcome this gap, we used nuclear Simple Sequence Repeats (nSSR) to study the patterns of genetic diversity and population structure of *B. distachyon* in 14 populations sampled across the Iberian Peninsula (Spain), one of its best known areas.

**Results:**

We found very low levels of genetic diversity, allelic number and heterozygosity in *B. distachyon*, congruent with a highly selfing system. Our results indicate the existence of at least three genetic clusters providing additional evidence for the existence of a significant genetic structure in the Iberian Peninsula and supporting this geographical area as an important genetic reservoir. Several hotspots of genetic diversity were detected and populations growing on basic soils were significantly more diverse than those growing in acidic soils. A partial Mantel test confirmed a statistically significant Isolation-By-Distance (IBD) among all studied populations, as well as a statistically significant Isolation-By-Environment (IBE) revealing the presence of environmental-driven isolation as one explanation for the genetic patterns found in the Iberian Peninsula.

**Conclusions:**

The finding of higher genetic diversity in eastern Iberian populations occurring in basic soils suggests that these populations can be better adapted than those occurring in western areas of the Iberian Peninsula where the soils are more acidic and accumulate toxic Al ions. This suggests that the western Iberian acidic soils might prevent the establishment of Al-sensitive *B. distachyon* populations, potentially causing the existence of more genetically depauperated individuals.

**Electronic supplementary material:**

The online version of this article (doi:10.1186/s12862-017-0996-x) contains supplementary material, which is available to authorized users.

## Background

During the last decade *Brachypodium distachyon* (L.) P. Beauv. has become one of the most important model systems for functional genomic studies of temperate cereals and forage grasses and for bioenergy crops [1, 2]. The diploid *Brachypodium distachyon* shows a short generation time (annual life cycle), one of the smallest genomes among grass species (272 Mbp in five chromosomes) and it is a highly selfing plant that can easily be grown under controlled conditions [2]. The phylogenetic position of *B. distachyon* reinforces its importance as a model system since it is nested within tribe Brachypodieae (formed by exclusively by the genus *Brachypodium* P. Beauv.), and its sister relationship to the ‘core pooids’, a recently evolved lineage of subfamily Pooideae Benth. (Poaceae Barnhart), composed by the four grass tribes that encompass the vast majority of domesticated cool season cereal grain, forage, and turf crops [3, 4].

A high-quality reference genome of *B. distachyon* (based on the diploid inbred line Bd21) is already available [2] and significant investments have been further made in developing and using *Brachypodium* as a model system to learn the genetic mechanisms controlling relevant traits such as cell wall composition, biomass yield, abiotic and biotic stress tolerance, grain development and other features relevant to biomass crop development [3, 5–10]. For instance, candidate genes identified from C_4_ grasses that are emerging biomass crops (e.g., maize, sorghum) are being introduced into the temperate C_3_-plant *B. distachyon* with the aim of changing its photosynthetic characteristics since the C_4_ photosynthetic pathway is generally more efficient under hot and dry conditions [11].

An important key resource essential in any model system is the existence of germplasm collections and inbred lines that reflect traits of interest, as well as its natural genetic variation, which is considered to be the main resource for evolutionary change and for the adaptive potential of a species [3, 12–14]. For instance, in the model plant *Arabidopsis thaliana*, molecular analysis of its natural genetic variation has not only discovered a correlation between the allelic variation of known genes and the phenotypic variation of the species, but has also led to the discovery of novel genes [15]. However, despite all genomic progresses in *B. distachyon* and the fact that it is widely spread across the Mediterranean area [16–19], information about its natural genetic diversity remains scarce. For instance, the first large collection of inbred diploid *B. distachyon* lines was developed from samples collected across the same geographical area (Turkey) but revealed a considerable level of inter-population genetic diversity despite the predominance of homozygous individuals in most populations [20, 21]. A recent study using genotyping-by-sequencing (GBS) of 84 new accessions of *B. distachyon* plus its close relatives (three accessions of *B. stacei* Catalán, Joch. Müll., Mur & Langdon and seven of *B. hybridum* Catalán, Joch. Müll., Hasterok & Jenkins) across its wide circum-Mediterranean native geographic range (e. g., Albania, Armenia, Georgia, Italy, Spain and Turkey) revealed low levels of gene flow, confirming the highly selfing nature of this species and detecting three distinct genetic groups in *B. distachyon* across the Mediterranean populations sampled [22]. Unexpectedly, those genetic groups were not correlated with the geographical origin of the accessions but rather with differences in flowering time, according to the common garden experiment performed [22]. The finding of highly diverged genetic groups is intriguing since individuals clustering to different groups were collected in the same or nearby localities [22]. This would mean that individuals growing in the same locality and under the same environmental conditions could have strong differences in flowering times, creating a barrier to gene flow with their close neighboring individuals [3, 21]. Moreover, it clearly reflects that more studies are needed to understand the natural genetic diversity and genetic structure of *B. distachyon* populations. This information is also crucial to establish efficient germplasm collections and reference lines for the ongoing genomic studies that are being developed by the International *Brachypodium* Initiative (e.g., http://jgi.doe.gov/our-science/science-programs/plant-genomics/brachypodium/brachypodium-t-dna-collection/; http://archive.gramene.org/species/brachypodium/brachypodium_intro.html), especially because there is evidence that annual *Brachypodium* species are ecologically differentiated [12, 23]. The diploid *B. distachyon* usually grows in wet habitats with attenuated summer drought while the allotetraploid *B. hybridum* is generally found in dry habitats with a predictable summer drought period [23]; the allotetraploid is also more efficient in its water use than the close-related diploid *B. distachyon* [12]. Drought-escape strategy (i.e., early flowering) to cope with water stress was found to affect genetic diversity in the studied *B. hybridum* populations but not in *B. distachyon* [13]. However, the potential influence of other environmental factors on the genetic diversity of the annual *Brachypodium* species and populations is still unknown. For instance, soil pH seems to be related to the ecological adaptation of some annual *Brachypodium* populations to acidic substrates [24]. Under acidic conditions (pH < 5.0), the soils can accumulate solubilized Aluminum (Al) ion, mostly as a mononuclear cation (Al^3+^), which is phytotoxic to most herbaceous plants even at low concentration [24, 25]. *Brachypodium distachyon* is mostly an Al-sensitive plant in contrast to its derived allotetraploid species *B. hybridum*, which shows both Al-tolerant and Al-sensitive populations [24].

Here, we studied the natural genetic diversity of *B. distachyon* across 14 populations collected in the Iberian Peninsula to create and characterize a future diverse collection of inbred lines, available to *Brachypodium* researchers. This is the best studied Mediterranean area due to several previous works, which allowed us to correctly separate the diploid *B. distachyon* (2*n* = 10) from its close diploid relative *B. stacei* (2*n* = 20) and from their derived allotetraploid *B. hybridum* (2*n* = 30), which were until recently misinterpreted as a single complex species under *B. distachyon* [16–19, 23]. In this study we specifically asked: (1) Is genetic diversity uniform across the Iberian populations of *B. distachyon*? (2) Is homozygosis predominant within populations as expected in a highly selfing plant? (3) How are populations of *B. distachyon* structured genetically? (4) Is there a correlation between genetic diversity and climatic, geographic or other ecological factors such as soil pH?

## Methods

### Population sampling, DNA extraction and nSSR amplification

**Fig. 1:**
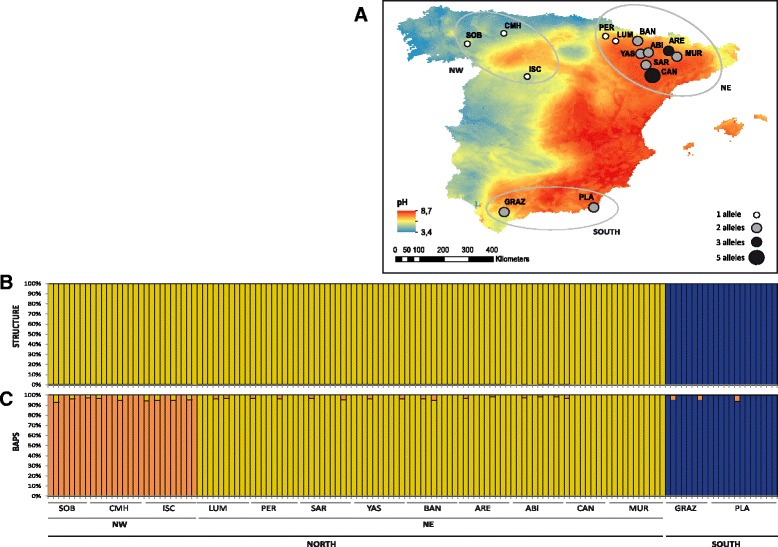
**a**: Soil pH and collection localities of *B. distachyon* populations in North-West (NW), North-East (NE) and South (S) Spain. Circles shown the number of unique alleles found in each population as indicated in the chart. **b**, **c** Population structure of *B. distachyon* based on 10 nSSRs and using the best assignment result retrieved by STRUCTURE (B: *K* = 2) and BAPS (C: *K* = 3). Each individual is represented by a thin vertical line divided into *K* colored segments that represent the individual’s estimated membership fractions in *K* clusters. The different population codes (see Table 1) and geographic areas are labeled below the graph

A total of 137 individuals were sampled across 14 populations of *B. distachyon* covering the whole distribution range of this species within the Iberian Peninsula (Fig. 1a). In each population, 8–13 individuals were randomly collected with a minimum sampling distance of 10 m. Individual plants from Northeast (NE) Iberian populations LUM, PER, BAN, ARE, YAS, ABI, SAR, CAN and MUR were sampled in the wild, whereas individuals from Northwest (NW) Iberian populations SOB, CMH and ISC and South (S) Iberian populations PLA and GRAZ are first generation seed-germinated selfed plants (S1), each of them obtained from a different wild mother plant. Sampling sizes, locations and geographic coordinates of each population studied are shown in Table 1. Because in some populations *B. distachyon* can morphologically be confounded with the hybrid *B. hybridum*, the identity of the samples was confirmed through DAPI-staining chromosome counting of the studied materials, coupled with barcoding markers, as indicated in [18]. Fresh leaves were collected for each individual, dried in silica gel and stored at −20 °C until DNA was extracted. Individual samples were stored in the DNA bank of the Bioflora group at the University of Zaragoza in Spain (http://www.bifi.es/bioflora/) and voucher specimens were deposited in the JACA herbarium in Spain (http://herbario.ipe.csic.es/).

**Table 1:** Sampled populations of *Brachypodium distachyon* sorted by geographical area

Locality	Code	*N*	Latitude (N)	Longitude (W)	Altitude (m.a.s.l)*	H_o_	H_e_	N_A_	A_R_	F_IS_	s	Exclusive genotypes	pH
NW Spain													
Ourense, Sobrado	SOB	9	42.53167	−6.85194	464	0.000	0.000	10	1.000	-	-	1 (11.1%)	5.36
Leon, Campohermoso	CMH	10	42.85472	−5.43472	1074	0.000	0.000	10	1.000	-	-	1 (10.0%)	6.67
Valladolid, Iscar	ISC	8	41.37	−4.53833	828	0.000	0.000	10	1.000	-	-	1 (12.5%)	6.67
NE Spain													
Navarra, Lumbier, Foz	LUM	10	42.63651	−1.30484	434	0.000	0.000	10	1.000	-	-	1 (10.0%)	8.01
Navarra, Puerto del Perdon	PER	10	42.73703	−1.74952	736	0.000	0.000	10	1.000	-	-	1 (10.0%)	8.01
Huesca, Sariñena Laguna	SAR	10	41.78622	−0.18278	292	0.000	0.032	11	1.100	1.000	1.000	2 (20.0%)	8.36
Huesca, Yaso, Sierra de Guara	YAS	10	42.2024	−0.12232	731	0.000	0.032	11	1.100	1.000	1.000	2 (20.0%)	8.36
Huesca, Jaca, Banaguas	BAN	10	42.58063	−0.5799	822	0.000	0.018	11	1.100	1.000	1.000	2 (20.0%)	8.36
Huesca, Aren	ARE	10	42.256944	0.72814	681	0.000	0.060	12	1.200	1.000	1.000	3 (30.0%)	8.36
Huesca, Abizanda, Barranco Mallo	ABI	10	42.195	−0.207	382	0.000	0.018	11	1.100	1.000	1.000	2 (20.0%)	8.36
Zaragoza, Candasnos	CAN	9	41.4651	0.0176	206	0.033	0.104	13	1.300	0.711	0.831	5 (55.5%)	8.27
Lleida, Castillo de Mur	MUR	10	42.09763	0.8775	487	0.000	0.018	11	1.100	1.000	1.000	2 (20.0%)	7.87
S Spain													
Cadiz, Grazalema	GRAZ	8	36.75583	−5.44167	1103	0.000	0.022	11	1.100	1.000*	1.000	2 (25.0%)	8.09
Almeria, Playazo-Rodalquilar	PLA	13	36.8609867	2.0007	11	0.000	0.014	11	1.100	1.000*	1.000	2 (15.4%)	8.59

The location, population code, number of plants genotyped (*N*), latitude, longitude, altitude, soil pH, mean observed heterozygosity (*H*_*o*_) and expected heterozygosity (*H*_*e*_), total number of alleles (*N*_*A*_), mean allelic richness (*A*_*R*_), inbreeding coefficient (F_IS_), selfing rate (*s*), and number of exclusive genotypes (%. between parenthesis) are shown. Asterisks indicate F_IS_ values (range 0–1) deviating from Hardy-Weinberg Equilibrium (HWE). Soil pH values were retrieved from [51]

*m.a.s.l: meters above sea level

Total genomic DNA was extracted using the DNeasy Plant Mini Kit (Qiagen, Valencia, CA, USA) according to the manufacturer’s protocol. The 137 samples used in this study were genotyped at 10 polymorphic nuclear simple sequence repeats (nSSRs) previously developed for *B. distachyon* (ALB006, ALB022, ALB040, ALB050, ALB086, ALB087, ALB139, ALB165, ALB181 and ALB311; [21]) and following the procedures outlined in [26]. Based on an initial survey, we selected these ten nSSR markers since they produced robust highly polymorphic amplified bands among the entire collection of our *B. distachyon* samples. Despite being a model system with a variable set of genomic resources, new genomic population methods such as Genotyping-by-Sequencing (GBS, [22]) or restriction site associated DNA sequencing (RAD-Seq; Lopez-Alvarez & Catalan, *unpub. Data*) have only recently started to become available for *B. distachyon*. Although these next-generation sequencing (NGS) techniques will probably be predominant in next years, SSRs still have advantages if they are genetically informative, like previously reported in *Brachypodium distachyon* [21] an in its close annual Mediterranean congeners *B. stacei* [26] and *B. hybridum* [27], as well as in the Eurasian perennial *B. sylvaticum* [28, 29]. Also, the number of biases in a SSR study might be much lower than using NGS methods since each locus can be manually genotyped reducing errors [30].

Amplifications were carried out in a final volume of 10 μl volume containing between 0.1 and 0.2 μl of each 10 M diluted primer (forward and reverse), 5 μl PCR Master Mix (QIAGEN) and 2.5 μl DNA. The polymerase chain reactions (PCR) were carried out in a final volume of 7.5 μl on a GeneAmp PCR System 9700 thermocycler with a thermal profile consisting of a 4-min initial denaturation step at 95 °C followed by 35 cycles of 30 s at 95 °C, 30 s at 55 °C and 1 min at 72 °C. A final 72 °C extension step of 30 min was included to promote non-templated nucleotide addition at the 3’end of the PCR product. Multiplexed PCR products were genotyped on an Applied Biosystems 3130XL Genetic Analyzer using 2 μl of amplified DNA, 12 μl of Hi-Di formamide and 0.4 μl of GeneScan-500 (LIZ) size standard (Applied Biosystem). Allele sizes were determined using Peak Scanner version 1.0 (Life Technologies) and revised manually. The list of individuals genotyped is shown in Additional file 1: Table S1. Allelic sizes ranged within the expected values obtained for these markers in other genetic studies of *B. distachyon* [21, 26, 31]*.* Within each population, all loci were checked for the presence of null alleles using MICRO-CHECKER v.2.2.3 [32].

### Genetic diversity and selfing

Genetic variation was calculated per locus and population using the following standard genetic indices computed using FSTAT 2.9.3.2 [33]: total number of alleles (Na), allelic richness (A_R_), observed within population Nei’s heterozygosity (H_o_), expected within population Nei’s heterozygosity (H_s_), expected Nei’s heterozygosity within the total population (H_T_), Nei’s measure of genetic differentiation (G_st_), and inbreeding coefficient (F_IS_). F_IS_ was also estimated using the Bayesian procedure implemented in INEst 2.0 [34] that is robust to the presence of null alleles. Posterior distribution was based on 300,000 steps, sampling every 100 steps and discarding the first 30,000 steps as burn-in. In order to understand the importance of inbreeding in our dataset we compared the full model (nfb) with the model including only null alleles (nb). The best model was chosen based on the Deviance Information Criterion (DIC; cf. [35]).

### Genetic structure and differentiation

The Bayesian program STRUCTURE v.2.3.4 [36] was used to test whether any discrete genetic structure exists among the populations sampled. The analysis was performed assuming a number of clusters from *K* = 1 to *K* = 17, with 10 repetitions per *K*. Models were run assuming ancestral admixture and correlated allele frequencies with 50,000 burn-in steps, followed by run lengths of 300,000 interactions for each K. The optimum *K* was determined using STRUCTURE HARVESTER [37], which identifies the optimal *K* based both on the posterior probability of the data for a given K and the ∆K [38]. To correctly assess the membership proportions (q values) for clusters identified in STRUCTURE, the results of the replicates at the best-fit *K* were post-processed using CLUMPP 1.1.2 [39]. BAPS v.5.2 [40] was also used to estimate population structure of *B. distachyon*. In contrast to STRUCTURE, BAPS determines optimal partitions for each candidate K-value and merges the results according the log-likelihood values to determine the best *K*-value. Analyses in BAPS were done at the level of individuals using the models without spatial information and by selecting 1 to 17 as possible K-values. Ten repetitions were performed for each *K*. POPULATION 1.2 [41] was used to calculate the Nei’s genetic distance (Da; [42]) among individuals and to construct an unrooted neighbor-joining tree with 1000 bootstrap replicates. A Principal Components Analysis (PCoA) was also constructed in GenAlEx6 [43] to detect the genetic relatedness among individuals based on Nei’s genetic distance.

Standard and hierarchical analysis of molecular variance (AMOVA) were used to quantify the partitioning of genetic variance within and among the following hierarchal levels: among all populations, between N and S populations, between NE and NW populations, and among NW, NE and S populations. In each analysis, variance was quantified among groups, among locations within groups and within sampling locations. Each AMOVA was run with 10,000 permutations at 0.95 significance levels. The analysis was performed in ARLEQUIN 3.5.1.3 [44].

Pairwise genetic distances between populations were calculated using three metrics in order to cover a range of evolutionary assumptions concerning the relationships between populations [45]. We computed pairwise genetic distances assuming both the infinite alleles model (IAM), e. g., Da distance [42], and the stepwise mutation model (SMM), e. g., Average Square Distance (ASD; [46]), as implemented in POPULATION 1.2 [41], and pairwise linearized FST value distances, e.g., FST/(1 - FST [42] between populations as implemented in GENPOP 3.3 [47]. Isolation by distance (IBD) was assessed through the correlation between the three genetic distance matrices and a matrix of pairwise geographical distances between populations computed with GEOGRAPHIC DISTANCE MATRIX GENERATOR v1.2.3 [48]. Significance was tested with Mantel tests [49] with 1000 permutations using NTSYSpc v. 2.11a [50].

### Association between genetic diversity and ecological variables

Using the Pearson correlation coefficient, we tested the degree of association between five genetic diversity parameters (A_R_, N_a_, H_o_, H_e_ and F_IS_), and latitude, longitude, altitude plus 19 Bioclimatic variables (Bioclim) previously used in the distribution modeling of annual *Brachypodium* species ([23]; Additional file 2: Table S2) and downloaded from Worldclim- Global Climate Data (http://www.worldclim.org) at a scale of 30 arc-seconds. Soil pH values were retrieved from [51]. A correlation analysis between Bioclim variables was first conducted for avoiding variable redundancy [50]: eight variables were considered correlated (Pearson coefficient: *R* ≥ 0.95; Additional file 2: Table S2) and were removed from further analyses. Environmental distances between populations were then estimated to test isolation by environment (IBE) using the 11 Bioclim variables that did not covary significantly. As environmental and geographical distances were significantly correlated (*R* = 0.35, *P* = 0.001), IBE was tested using a partial Mantel test with 10,000 permutations as implemented in R using the ‘*ecodist*’ package [52]. This test allows discriminating unambiguously between environmental and geographical factors in the correlation structure with genetic variables. Pearson correlation analyses were implemented using the SPSS statistical software package 16.0 (SPSS Inc., Chicago, IL, USA). Holm-Bonferroni corrections were conducted using the R statistical software package (R Development Core Team 2013) to avoid type I error inherent in multiple comparisons [52]. The significance level was *P* < 0.05.

## Results

### Genetic diversity and selfing

The total number of alleles per locus varied between 14 recorded in 6 of the 10 loci studied (*ALB022, ALB086, ALB087, ALB139, ALB181, ALB311*) and 19 alleles (*ALB050*) (Table 2). Allelic richness per locus varied between 1.983 (*ALB181*) and 4.838 (*ALB050*). Null allele frequencies calculated with INEst were always very low with an average of 0.003 across loci (Table 2) although MICROCHECKER did not detect any null alleles. Nei’s observed heterozygosity per locus was recorded as 0 except in locus *ALB024*, which had a value of 0.024, while overall within population Nei’s expected heterozygosity varied between 0 and 0.099 (Table 2). However, the expected Nei’s heterozygosity within the total population per locus exhibited generally a higher value, reaching an average of 0.507. According to the Nei’s measure of genetic differentiation (G_st_), the estimated divergence of populations per locus varied from 0.819 (*ALB040*) to 1, in 6 of the 10 analyzed loci (Table 2). The inbreeding coefficient F_IS_ had an overall value of 0.922 although it was fixed at 1, in 9 of the 10 loci analyzed (all except locus *ALB040*). Similar values were retrieved when F_IS_ was calculated in INEst though values were higher for locus *ALB040* (Table 2). Results from Bayesian analyses implemented in INEst revealed that only inbreeding contributed to the excessive homozogosity since this model (DICnfb: 2578.593) was preferred over the model that included only null alleles (DICnb: 3683.439) based on the DIC criterion.

**Table 2:** Characteristics of the nSSRs markers used in the Iberian populations of *Brachypodium distachyon*

Locus	Repeat motif	Allele size range (bp)	N_a_	p_null_	A_R_	H_o_	H_s_	H_T_	G_st_	F_IS_	F_IS_^§^
*ALB006*	(GT)15	360–374	16	0.00399	3.318	0.000	0.051	0.614	0.917	1.000^a^	1.000
*ALB022*	(CT)11	354–358	14	0.00464	2.909	0.000	0.000	0.520	1.000	1.000	1.000
*ALB040*	(CTT)8	176–182	15	0.00416	1.975	0.024	0.031	0.169	0.819	0.221^a^	0.871
*ALB050*	(GT)15	217–231	19	0.00311	4.838	0.000	0.072	0.794	0.910	1.000^a^	1.000
*ALB086*	(AAG)7	190–198	14	0.00434	2.909	0.000	0.000	0.520	1.000	1.000	1.000
*ALB087*	(AGC)7	192–202	14	0.00355	2.909	0.000	0.000	0.520	1.000	1.000	1.000
*ALB139*	(AGA)7	308–310	14	0.00495	1.999	0.000	0.000	0.459	1.000	1.000	1.000
*ALB165*	(ATA)12	173–201	18	0.00369	4.745	0.000	0.099	0.776	0.873	1.000^a^	1.000
*ALB181*	(AC)9	234–238	14	0.00437	1.983	0.000	0.000	0.337	1.000	1.000	1.000
*ALB311*	(GA)6	244–250	14	0.00152	2.649	0.000	0.000	0.357	1.000	1.000	1.000
*Overall*	-	-	15	0.00383	3.023	0.002	0.025	0.507	0.952	0.922	0.987

Na: total number of alleles; p_null_: average frequency of null alleles across populations; A_R_: average allelic richness; H_s_: expected within population Nei’s heterozygosity; H_o_: observed within population Nei’s heterozygosity; H_T_: expected Nei’s heterozygosity within the total population; G_st_: the Nei’s measure of genetic differentiation; F_IS_: inbreeding coefficient estimated in FSTAT (^a^ indicates values deviating from HWE); F_IS_^§^: inbreeding coefficient estimated using the Bayesian procedure implemented in INEst)

Within populations, observed heterozygosity was recorded as 0, in 13 out of the 14 populations analyzed except the NE Iberian population of CAN where it had a value of 0.033, whereas mean expected heterozygosity varied between 0 and 0.104 also in CAN (Table 1). The average number of alleles per population was 11, being a maximum of 13 recorded in CAN, which also showed the maximum value of allelic richness (Table 1). Due to the very low levels of heterozygous individuals found, most populations had a F_IS_ value of 1 (fixed homozygosis) except in the NE Iberian population of CAN where F_IS_ was estimated as 0.711 (Table 1). Therefore, the average rate of self-fertilization estimated for *B. distachyon* was very high, reaching an average of 98% considering all the populations studied (Table 1).

**Fig. 2:**
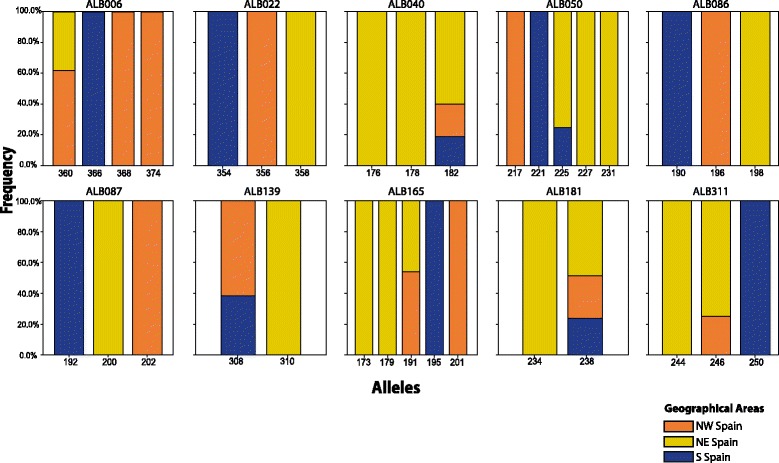
Frequency of the alleles found in *Brachypodium distachyon* across the three geographical areas sampled: North-West (NW), North-East (NE) and South (S) Spain. The x-axis indicates the allele size (see Additional file 1: Table S1)

Due to the high level of homozygosis (fixed alleles) observed in most populations, only 27 out of 137 genotyped individuals of *B. distachyon* (19.7%) exhibited a unique multi-locus genotype (Table 1). A relatively high number of unique multi-locus genotypes were found in the NE population of CAN. From the 14 sampled populations of *B. distachyon*, only 33 alleles were found in the 137 individuals studied (Fig. 2). Seven out of the 33 alleles were exclusively found in the southern Iberian populations, 7 only in the NW Iberian populations and 12 only in the NE Iberian populations. Only 7 alleles were shared between geographic regions: 3 between NW and NE Iberian populations, 1 between NW and S Iberian populations, 1 between NE and S Iberian populations, and 2 between the three regions (Fig. 2).

### Population genetic structure and differentiation

The optimal number of genetic clusters was found to be two by the Bayesian clustering program STRUCTURE that differentiated all North Iberian populations from the South Iberian populations of *B. distachyon* (Fig. 1b; Additional file 3: Fig. S1). This result was partially supported by the Bayesian BAPS analysis that further separated the northern populations into two segregated groups, suggesting an optimal clustering of populations into three genetic groups (NE Iberian, NW Iberian and S Iberian: Fig. 1c). These two programs detected no evidence of genetic admixture between the genetic clusters.

**Fig. 3:**
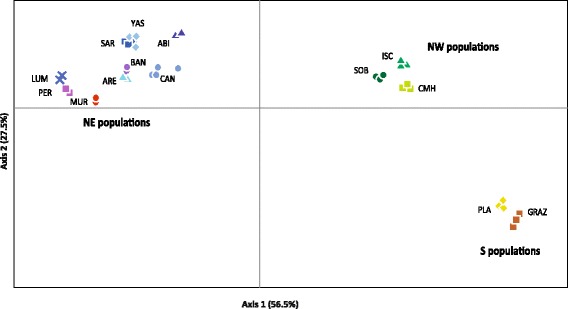
Principal Coordinate analysis (PCoA) of the studied *Brachypodium distachyon* samples using the scored nSSRs markers. Percentage of explained variance of each axis is given in parentheses. Population symbols and colours are shown in the chart. Population codes are indicated in Table 1. Please note that only a small number of individuals can be seen since most individuals bear identical alleles within each population

The PCoA spatially separated all populations analyzed into three main groups that clustered NW, NE and S Iberian populations (Fig. 3) being consistent with the genetic structure obtained from BAPS. NE Iberian and NW plus S Iberian populations clustered at both extremes of axis 1, which accumulated 56.1% of variance, while S Iberian populations separated from the NW populations at the negative extreme of axis 2, which accumulated 27.5% of variance (Fig. 3). Due to the high level of fixed alleles within each population, only a very low number of individuals are seen in the PCoA since most individuals bear identical alleles within a population.

**Fig. 4:**
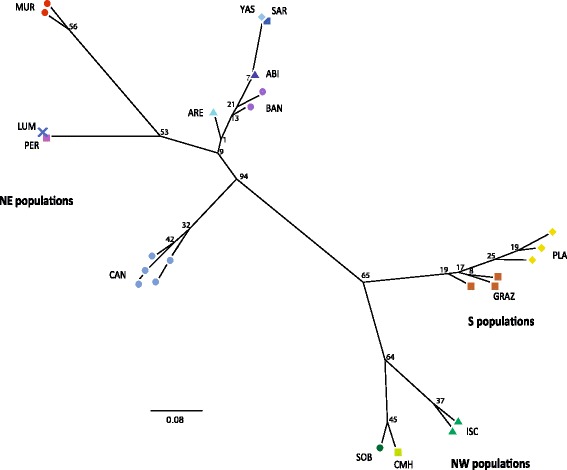
Unrooted neighbor-joining tree of the studied *Brachypodium distachyon* populations based on Nei’s Da genetic distance. Numbers associated with braches indicate bootstrap values based on 1000 replications. Populations symbols and colours followed the ones depicted in Fig. 3. Population codes are indicated in Table 1. Please note that only a small number of individuals can be seen since most individuals bear identical alleles within each population

The NJ separated all NE Iberian populations, which were grouped in a highly supported clade (94% bootstrap support value, BS), from NW and S populations that clustered in a moderately supported group (65% BS; Fig. 4). Within the last clade, the NW Iberian populations clustered in a group with 64% BS. The remaining sub-divisions found in the NJ tree correspond mainly to the populations sampled although BS values were always very low (<50%, Fig. 4). As mentioned above, only a small number of individuals can be seen in the NJ tree since most individuals within each population share the same alleles.

Genetic differentiation across all 14 populations was significantly high (AMOVA F_ST_ = 0.956, *P* < 0.00001). Overall, 96.9% and 3.1% of the genetic variation was attributed to variation among and within populations, respectively (Table 4). To further investigate the genetic differentiation between the geographical areas, a hierarchical AMOVA performed between N and S Iberian populations (matching the genetic boundary defined by STRUCTURE) attributed similar percentages of variation among groups (54%) and among populations within groups (43.7%; *P* < 0.00001, Table 3; F_ST_ = 0.942, F_SC_ = 0.973 and F_CT_ = 0.537). However, it was further exacerbated when performing the hierarchical AMOVA between the NE, NW and S Iberian populations (groups recovered by BAPS), which showed the highest partition of variance among groups (73.9%) and the lowest partition among populations within groups (24%, *P* < 0.00001, Table 4; F_IT_ = 0.869, F_SC_ = 0.921, F_CT_ = 0.739, F_IT_ = 0.997). These values were similar to those obtained in a restricted hierarchical AMOVA conducted only with northern Iberian populations (NE vs NW), where 70% and 26.7% of the total variance was distributed among groups and among populations within groups, respectively (*P* < 0.00001, Table 3; F_ST_ = 0.894, F_SC_ = 0.968 and F_CT_ = 0.701).

**Table 3:** Analysis of molecular variance (AMOVA) for 14 populations of *Brachypodium distachyon*

Source of variance	d.f.	Variance components	% Variance
All populations			
Among populations	13	2.64	95.92
Within populations	260	0.08	3.08
N vs. S populations			
Among groups	1	2.38	53.76
Among populations within groups	12	1.93	43.57
Within populations	260	0.11	2.67
NE vs. NW populations			
Among groups	1	2.74	70.18
Among populations within groups	10	1.04	26.66
Within populations	220	0.12	3.16
NE vs. NW vs S populations			
Among groups	2	2.99	73.91
Among populations within groups	11	0.97	24.02
Within populations	260	0.08	2.07

**Table 4:** Significance differences of the correlation analysis (corrected for multiple comparisons following the Bonferroni procedure) between geographical, soil pH and climatic factors and genetic diversity parameters: mean allelic richness (A_R_), mean observed heterozygosity (Ho) and expected heterozygosity (Hs) and inbreeding coefficient (F_IS_)

Parameter	Latitude	Longitude	Altitude	pH	BIO3	BIO8	BIO9
A_R_	−0.178	0.558*	−0.353	0.559*	−0.603*	0.699*	−0.560*
H_o_	0.002	0.771	−0.346	0.159	−0.240	0.386	−0.225
H_s_	−0.050	0.444	−0.313	0.435	−0.451	0.231	−0.278
N_a_	−0.178	0.558*	−0.353	0.549*	−0.529*	0.612*	−0.566*
F_IS_	−0.278	−0.062	0.349	0.023	−0.603*	0.699**	−0.560*

Only soil pH and the three significantly associated bioclimatic variables are shown. BIO3: Isothermality; BIO8: Mean Temperature of Wettest Quarter, BIO9: Mean Temperature of Driest Quarter

*P < 0.05, **P < 0.001

Correlation between each of the three assayed pairwise genetic distance metrics and pairwise geographical distances revealed significant evidence of IBD between all 14 populations analyzed (DA/geography, *r* = 0.843, *P* < 0.001; ADS/geography, *r* = 0.543, *P* < 0.001; linearized FST values/geography, *r* = 0.637, *P* = 0.001). Genetic distances based on the IAM (Da) showed a clustering of populations more congruent with geography than those based on the SMM (ADS), or the linearized Fst values.

### Association between genetic diversity and ecological variables

Two genetic diversity parameters (A_R_ and N_a_) were significantly and negatively associated with the longitude indicating a decrease in genetic diversity towards West (Fig. 1a); no association was found for the remaining genetic diversity indices (Table 4). The level of pH was significantly and positively associated with A_R_ and N_a_ since a higher allelic richness and a higher number of alleles were generally found in populations occurring in basic soils (Fig. 1a), located in the East. Of the 11 climatic variables analyzed, two were significantly negatively associated with A_R_, Na and F_IS_ (isothermality, BIO3; mean temperature of the driest quarter, BIO9) and one (mean temperature of the wettest quarter, BIO8) was positively associated with those genetic parameters (Table 4; Additional file 4: Fig. S2). Thus, the higher are the values of isothermality and temperature of the driest quarter, the lower is the genetic diversity measured in the populations sampled here; likewise, the higher the value of the temperature in the wettest quarter, the higher is the genetic diversity of the *B. distachyon* populations.

A partial Mantel test confirmed significant IBD among all studied populations (*R* = 0.15, *P* < 0.001), as well as significant IBE (*R* = 0.46, *P* < 0.05). This result indicates that by controlling geographical distance, pairwise differences in soil pH were positively associated to pairwise differences in genetic diversity, which could underlie an adaptive pattern to soil pH and, presumably, to Al sensitiveness. The level of significance in IBE was higher when performing correlation analyses between populations belonging to the NW genetic cluster versus populations from NE and S genetic clusters (*R* = 0.56, *P* < 0.001). No significant IBE was found when only the NE and S genetic cluster were analyzed (*R* = 0.38, *P* = 0.482).

## Discussion

### Very low heterozygosis across Iberian populations of *Brachypodium distachyon*

Our results indicate that the Spanish populations of *B. distachyon* are characterized by very low levels of genetic diversity within populations, as a consequence of a high heterozygote deficiency (Table 1; Additional file 1: Table S1). This could be partially a consequence of studying S1 individuals in S and NW Iberian populations; however, similar low genetic diversity and high heterozygote deficiency were observed among wild (non-S1) individuals in some NE Iberian populations (LUM, PER) (Table 1, Fig. 1a). An average of only two multi-locus genotypes was found, exceptionally reaching five in one population (Table 1). The number of alleles per population was also very low since only 33 unique alleles were retrieved among the 137 individuals of *B. distachyon* studied. The extreme low levels of observed heterozygosity (*H*_*o*_ = 0 in all populations except one; Table 1) point to high levels of inbreeding (F_IS_ = 1 in all populations except one; Table 1) and a strong selfing rate (*s* = 1 in all populations except one; Table 1).

These results are congruent with the highly selfing nature of this species like reported in other studies [3, 10, 21, 22]. Flowers of *B. distachyon* rarely open except under specific environmental conditions (warm, humid and full sun), although even in this case anthers dehisce to the stigmas under the fold of the palea causing primarily self-pollinations [21]. Close-related species, such as the sister species *B. stacei*, are also primarily selfing plants, though genetic diversity values suggest that it might outcross more often than *B. distachyon* [26]. For instance, selfing rates of Iberian, Balearic and Canarian *B. stacei* populations were estimated as 79% [26], which is lower than the ones reported here for Iberian *B. distachyon* populations. Also, the values of heterozygosity detected within populations were slightly higher in the diploid *B. stacei* (e. g., H_o_ = 0–0.058, H_e_ = 0–0.145; [26]) and contrastingly higher in the diploid and predominantly selfing perennial *B. sylvaticum* (H_o_ = 0.044–0.438, H_e_ = 0.076–0.592; [28, 29]) than the ones detected here in *B. distachyon* (H_o_ = 0–0.033, H_e_ = 0–0.104; Table 1).

Primarily selfing plants usually show high genetic differentiation among populations (e. g., [53, 54]). Selfing would explain the high levels of genetic differentiation and the very high fixation index found in *B. distachyon* (averaged F_ST_ = 0.956) since it leads to isolation and prevents the efficient flow of genes. Most genetic diversity (96.9%) was observed among populations and only 3.1% of genetic variation within populations (Table 4). Such differences were correlated with geographic distance suggesting the presence of barriers to gene flow between largely distant populations. Selfing indeed inhibits gene flow through pollen and exacerbates genetic differences and genetic structure [55], as found in our analysis. In *B. distachyon*, seed dispersal may also constrain effective gene flow since most seeds land very close to parental plants or are possibly dispersed by ants although within short dispersal distances from the mother plant [56]. The populations studied here are on the edge of the native distribution range of this species that occurs across the Mediterranean - SW Asian region [17–19, 23] but the observed low heterozygosity levels are similar to those found in populations located in other geographical areas, such as Turkey [21], as well as in other Mediterranean areas [22]. Thus, the extreme values of low heterozygosity seem to characterize this model species and it would be invaluable to study other populations to distinguish the influence of selfing from other processes that usually constrain the evolutionary success of populations (e. g., recurrent founder events [57]).

### Genetic boundaries in Iberian populations reflect their geographical origin

The results of BAPS (Fig. 1c) and PCoA analyses (Fig. 3), as well as the NJ tree (Fig. 4) and the hierarchical AMOVA with three geographical ranges (Table 3) suggest that the genetic structure of *B. distachyon* in Spain can be grouped (at least) in three clusters congruent with their geographical origin. NE, NW and S Iberian populations all formed separate and homogeneous groups except in the STRUCTURE analysis that clustered all NE and NW populations in one single group (Fig. 1b). Although more populations should be analyzed to verify the existence of further genetic groups in the Iberian Peninsula, our results provide evidence for the existence of a significant genetic structure of *B. distachyon* in Spain like previously suggested by a recent GBS study [22]. This study revealed a significant genetic boundary between NE and S Spanish populations, like the one reported here, although no NW Spanish populations were included in the study [22]. But contrary to the results of the GBS study where the genetic patterns of *B. distachyon* seemed to be primarily explained by differences in flowering time association, our results are better explained by the geographical origin of populations. Although we should keep in mind that PCR-based markers such as the one used here (nSSRs) and GBS techniques might reconstruct similar but slightly different stories [58], several other studies also reported the existence of differentiated genetic clusters in the Iberian geographical areas that we have studied (e. g., *Senecio boissieri* DC.: [59]; *Gentiana alpina* Vill., *Kernera saxatilis* (L.) Rchb. and *Silene rupestris* L.: [60]; *Ferula loscosii* Willk*.*: [61]; *Cheirolophus intybaceus* (Lam.) Dostál: [62]; *Gentiana lutea* L.: [63]).

Using the same set of nSSRs, we found two main genetic groups in the close relative *B. stacei* both distributed in southern Spain (S and SE Spain groups), from where it colonized the Mediterranean islands of Minorca and Majorca (SE Spanish group) and the oceanic Canary Islands (S Spanish group) apparently through different long distance dispersal (LDD) events [26]. The potential existence of different mechanisms for long distance dispersal of seeds (not related to geographical distances) was also invoked to explain the unexpected relationships of genetically similar but geographically disjunct *B. distachyon* lineages across the Mediterranean area [3, 18]. Here, the finding of essentially similar low within-population genetic diversities in *B. distachyon*, the low sharing of alleles between geographical regions, and the highly selfing nature of this species support vicariance rather than long-distance dispersal as our preferred explanation for the patterns found in our study.

### Ecological adaptations in *Brachypodium distachyon*

Besides historical factors (i.e., demography, glacial refugium), the genetic diversity of *B. distachyon* in the Iberian Peninsula seems to be also shaped by environmental isolation. Isolation by environment, in which genetic differentiation increases with environmental differences, independent of geographic distance, is one of the most important patterns that contribute to genetic divergence in nature [64]. However, a non-zero effect of IBE independent of IBD, like the one reported here has rarely been reported in other studies [65, 66]. Here, we found that the genetic diversity of *B. distachyon* is significantly positively associated with soil pH and the temperature of the wettest quarter, suggesting that the lower these variables are the less would be the genetic variability of the *B. distachyon* in the populations studied. By contrast, the significantly negative association found between the genetic diversity parameters and isothermality, as well as with the temperature of the driest quartersuggests contrary results.

In comparison to other areas, a high number of alleles and even some heterozygosity were found in the NE Iberian populations of *B. distachyon* (Pyrenees, pre-Pyrenees and the Ebro Valley) and in the S Iberian area (Table 1; Fig. 1). Indeed, the Pyrenees and pre-Pyrenees and the Betic ranges probably acted as major glacial refugia in southern Europe where many lineages came into contact [67–70]. These areas experienced several climatic changes given rise to a complex phylogeographic pattern of refugia within refugia (e.g., [69, 71–73]) that might also sustain the diversity of alleles found here. Despite the NE Iberian population were represented by wild individuals that could potentially have higher levels of genetic diversity than the S1 individuals of the S and NW Iberian populations, the high selfing nature of the species makes the sampling effect almost negligible. We should also point that our sampling is limited in the NW and S of the Iberian Peninsula but the geographic area where we are reporting the existence of a higher genetic diversity is congruent with the results found using GBS techniques and a wide sampling of *B. distachyon* throughout the Mediterranean basin [22]. In addition, our study has covered most areas of the Iberian Peninsula where the populations of *B. distachyon* grow and within the Iberian Peninsula our sampling reached novel geographic areas not included in the GBS study [22].

The highest diversity of *B. distachyon* found in the Ebro Valley, in a locality of very low altitude (CAN population), suggests that this area is also an important source of genetic diversity in this species, in accordance with several other phylogeographic studies (e.g., [74–76]). Palynological evidence also supports the Ebro Valley as an important glacial refugium during the last ice age and suggests the existence of a diverse composition of species in these glacial steppes [77–79]. In contrast to the strong topographic feature of the Pyrenees with peaks up to 3404 m a.s.l., and a general Eurosiberian climate (becoming more Mediterranean towards the east), with cold winters and heavily rainfall throughout the year, the Ebro Valley is characterized by a continental Mediterranean climate with low rainfall (300–350 mm/yr), high insolation and evapotranspiration (1000–1500 mm/yr), and the prevalence of strong, dry north-westerly winds where steppe grassland species predominate more often than in the Pyrenees [80]. The number of alleles of *B. distachyon* in the Ebro Valley and its sharing with the populations of the Pyrenees and pre-Pyrenees range suggest that this species could have expanded from this refugium through the NE Spanish Mountains explaining the low number of alleles found in other populations.

The finding of higher allelic richness and higher number of alleles in populations occurring in basic soils suggests that these populations might be better adapted than those occurring in western areas of the Iberian Peninsula were the soils are more acidic (Fig. 1a), and therefore could accumulate Al ions causing toxicity in most plants, including *B. distachyon*, which is mostly an Al-sensitive plant [24]. Thus, this could indicate that western Iberian acidic soils might prevent establishment and expansion of Al-sensitive *B. distachyon* populations, potentially causing the existence of more genetically depauperated individuals. Nevertheless, while low soil pH and the resulting increased Al-induced phytotoxicity could explain the low genetic diversity found here, the data in this study is insufficient to support a causal connection and this hypothesis should be tested experimentally. It is also worth noting that our soil pH levels were not measured during our sampling but rather taken from a publication. Although pH levels might be stable over time, this argues for caution when interpreting the accuracy of the statistical correlations found here.

### Progress towards new genomic initiatives in *Brachypodium distachyon* and current limitations

The finding of a higher level of genetic variability and adaptation of *B. distachyon* to basic soils is promising within an agricultural context where tree iron chlorosis is a problem in some basic soils, which could be alleviated by grass covers, like those of annual *Brachypodium* species [81]. This species and *B. hybridum* can protect the soil from being eroded [81] and are therefore suitable grass cover crops to olive grooves, vineyards and dry fruit croplands [81, 82]. Due to the high degree of homozygosity, obtaining inbreeding lines of *B. distachyon* can be easily done even under laboratory conditions allowing the rapid generation of reference and cultivated lines. A large and diverse germplasm collection of *B. distachyon* has now been assembled and it is freely available for the research community but new genetic studies continue to demonstrate novel unsuspected geographical areas of genetic diversity like the ones reported here. This demonstrates that more population genetic studies are needed to fully uncover the genetic diversity of this species. For instance, our knowledge concerning Central and Eastern Mediterranean and SW Asian populations is still limited (but see [14, 21]) and more studies are necessary to understand the genetic structure of this species across its native Mediterranean distribution. It would also be important to compare the native Mediterranean populations of *B. distachyon* to the ones introduced in other areas (e. g., Australia, J. Borewitz & J. Streitch, *pers. com.*) since although genetic diversity is lower in introduced areas, invasiveness might have also triggered the activation of new allelic variants in this species.

## Additional files

Additional file 1: Table S1. Genotypes of the studied Spanish *Brachypodium distachyon* individuals based on nSSR analysis (TXT 12 kb)

Additional file 2: Table S2. Description of the bioclimatic worldclim layers (http://www.worldclim.org/bioclim) used in the correlation analysis (XLSX 10 kb)

Additional file 3: Figure S1. STRUCTURE analysis of *Brachypodium distachyon* in Spain. (A) Mean log probability of data LnP(D) over 10 runs for each K value as a function of K (error bars represent standard deviation). (B) Evanno’s ad hoc statistic; DK as a function of K (PDF 376 kb)

Additional file 4: Figure S2. Bioclimatic variables significantly associated to genetic diversity in *Brachypodium distachyon*. A. BIO3. B. BIO8. C. BIO9 (PDF 9255 kb)
