# Supplementary figures and images for: Environmental isolation explains Iberian genetic diversity in the highly homozygous model grass Brachypodium distachyon
Source: BMC Evol Biol. 2017 Jun 15;17:139. doi: 10.1186/s12862-017-0996-x (PMC5472904; doi:10.1186/s12862-017-0996-x)

A

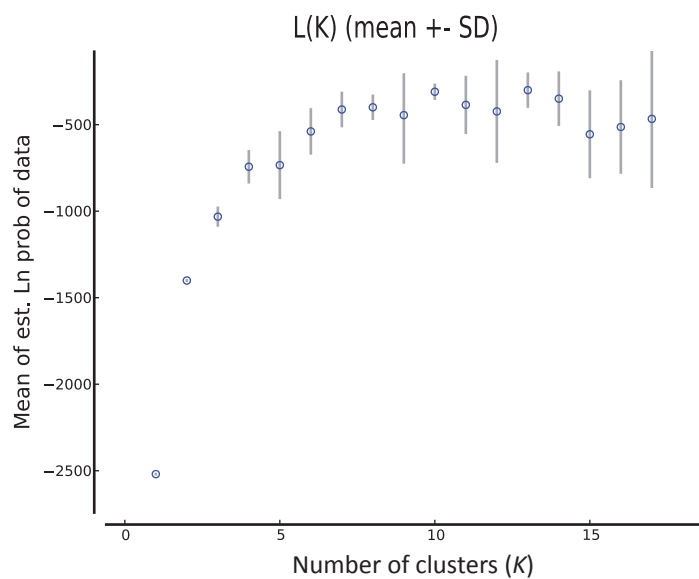

B

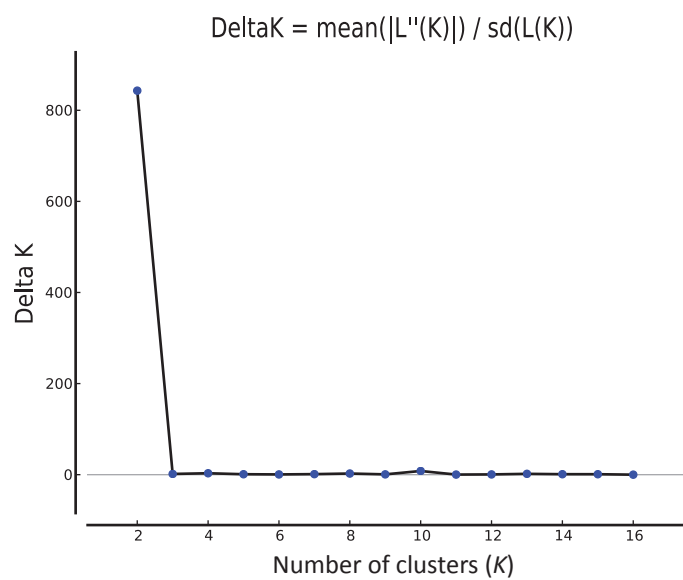

Supplement: Supplementary file 3 — STRUCTURE analysis of Brachypodium distachyon in Spain. (A) Mean log probability of data LnP(D) over 10 runs for each K value as a function of K (error bars represent standard deviation). (B) Evanno’s ad hoc statistic; DK as a function of K (PDF 376 kb) [file 12862_2017_996_MOESM3_ESM.pdf]

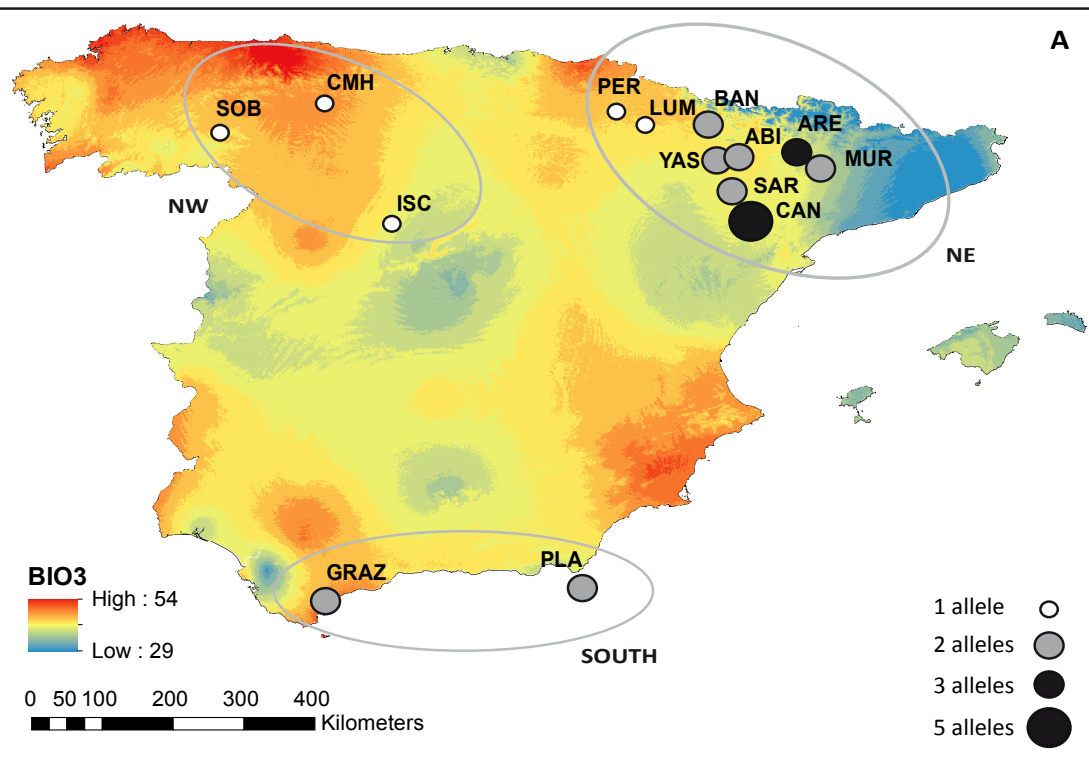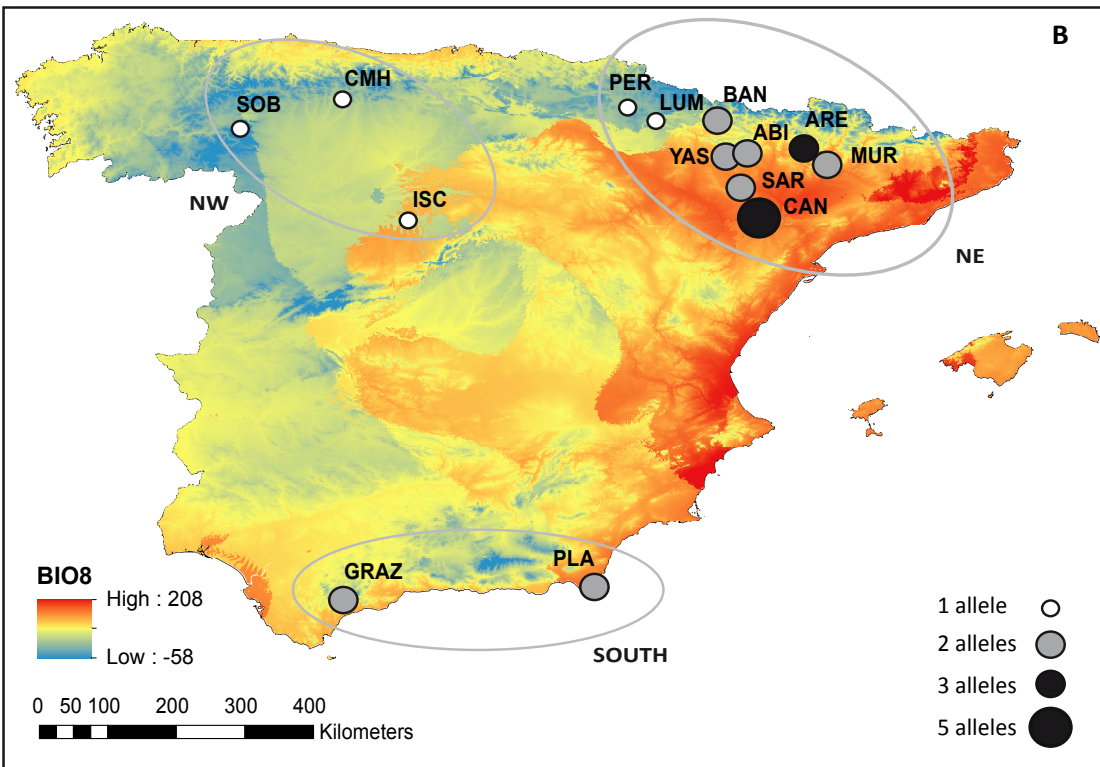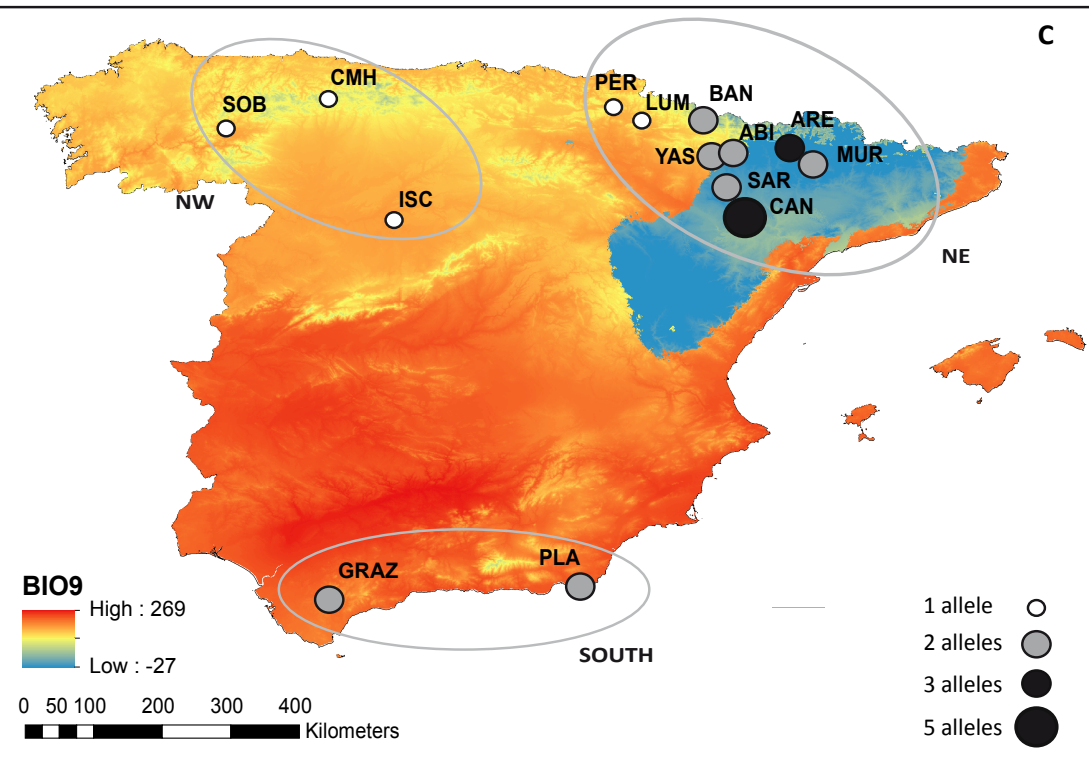

Supplement: Supplementary file 4 — Bioclimatic variables significantly associated to genetic diversity in Brachypodium distachyon. A. BIO3. B. BIO8. C. BIO9 (PDF 9255 kb) [file 12862_2017_996_MOESM4_ESM.pdf]
